# Determinant Factors Associated with Occurrence of Tuberculosis among Adult People Living with HIV after Antiretroviral Treatment Initiation in Addis Ababa, Ethiopia: A Case Control Study

**DOI:** 10.1371/journal.pone.0064488

**Published:** 2013-05-21

**Authors:** Kelemu Tilahun Kibret, Alemayehu Worku Yalew, Belaineh Girma Belaineh, Muluken Melese Asres

**Affiliations:** 1 Department of Public Health, College of Medical and Health Science, Wollega University, Nekemte, Ethiopia; 2 School of Public Health, Addis Ababa University, Addis Ababa, Ethiopia; 3 Help Ethiopia Address Low TB Performance (HEAL TB) Project, USAID/Management Sciences for Health (MSH), Addis Ababa, Ethiopia; Public Health Agency of Barcelona, Spain

## Abstract

**Introduction:**

Tuberculosis (TB) is a leading morbidity and mortality, and the first presenting sign in majority of people living with Human Immune deficiency Virus (PLWH). Determinants of active TB among HIV patients on anti retroviral treatment (ART) are not well described in resource limited settings. The aim of this study was to assess determinant factors for the occurrence of TB among people living with HIV after ART initiation in public hospitals and health centers in Addis Ababa, Ethiopia.

**Methods and Findings:**

A case control study was conducted from December 2011 to February 2012 in 2 public hospitals and 13 health centers in Addis Ababa. The study population consisted of 204 cases and 409 controls. Cases were adult people living with HIV who developed TB after ART initiation and controls were adult people living with HIV who did not develop TB after ART initiation. An interviewer administered structured questionnaire was used to collect information.

After adjustment for potential confounders, presence of isoniazid prophylaxis (adjusted odd ratio [AOR] 0.35, 95% confidence interval [CI] 0.125, 0.69) and cotrimoxazole prophylaxis (AOR = 0.19; 95% CI: 0.06, 0.62) had protective benefit against risk of TB. In contrary, bedridden (AOR = 9.36; 95% CI: 3.39, 25.85), having World Health Organization (WHO) clinical stage III/IV (AOR = 3.40; 95% CI: 1.69, 6.87) and hemoglobin level <10 mg/dl (AOR = 7.43; 95% CI; 3.04, 18.31) at enrollment to ART care were predictors for increased risk of tuberculosis in PLWH after ART initiation.

**Conclusion:**

Increasing coverage of isoniazid preventive therapy and cotrimoxazole preventive therapy reduced risk of TB among HIV patients who started treatment. All PLWH should be screened for TB, but for patients who have advanced disease condition (WHO clinical stage III/IV, being bedridden and having hemoglobin level <10 mg/dl) intensified screening is highly recommended during treatment follow up.

## Introduction

In high HIV prevalence population, tuberculosis (TB) is a leading cause of morbidity and mortality, and the first presenting sign in the majority of acquired immune deficiency syndrome (AIDS) patients [1], [2]. It is also the most common presenting illness among people living with HIV, including those who are taking antiretroviral treatment (ART) [3]–[5]. Despite major reductions with ART, however, risk of TB remains high in Africa [6].

According to the 2012 World Health Organization (WHO) global TB control report, Ethiopia ranks 8^th^ among the 22 high-burden countries in the world and the prevalence rate of TB including HIV positive TB (HIV+TB) is 237 per 1000,000 population with incidence rate of 258/100000 in 2011. There were 8% of HIV+TB patients in the country. The incidence rate of HIV+TB patients is 45/100000 [7] and number of TB case is more likely to increase in the country as HIV/AIDS epidemic expands [8].

The life time risk of developing active TB in HIV-negative individuals is approximately 10%, but the annual risk among HIV-infected patients is ∼10%, while the lifetime risk approaches 50% among them [9]. It is estimated that about one-third of people with HIV are also infected with TB [1]. Even though ART is known to decrease incidence of TB, still studies have reported TB incidence from HIV patients on ART [10]–[15].

In developing countries incidence of TB occurrence has been associated with factors like socio-economic [16]–[21], lifestyle/habits [17], [21], [22], clinical [16], [23]–[27], laboratory [10], [16], [19], [28], [29] and other co-morbidities, example, diabetes [30]. Many patients either have a history of TB when they start ART, or they develop TB while receiving ART in the developing world [6]. It has not been well delineated what factors influence the development of TB in patients on ART [23]. In sub-Saharan Africa including Ethiopia, the incidence of tuberculosis in adults receiving highly active antiretroviral therapy (HAART) is higher than in HIV-negative adults [3]. Studies on risk factors of TB were done in the general population but determinants of active TB among HIV patients are not well described in resource limited settings. There are no enough studies in Ethiopia on factors associated with development of TB among HIV infected patients who started ART. This study assessed the determinant factors for the occurrence of TB in people living with HIV (PLWHIV) who were already enrolled on ART in public hospitals and health centers, Addis Ababa.

## Methods

From December 2011 to February 2012 this case control study was conducted in two hospitals and thirteen health centers in Addis Ababa, the capital city of Ethiopia and seat of African Union & Economic Commission for Africa.

Cases were defined as adult people living with HIV who developed TB after ART initiation and on anti TB treatment in the last 6 months before data collection and controls were adults living with HIV who did not develop TB after ART initiation. Diagnosis of TB in HIV-positive patients was made based on the national TB guideline [31].

**Smear positive pulmonary tuberculosis (PTB+)** diagnosed if one sputum smear examination positive for Acid Fast Bacilli (AFB) by direct microscopy, **and** laboratory confirmation of HIV infection. And **smear negative pulmonary tuberculosis (PTB−)** diagnosed if at least two sputum specimens negative for AFB and radio graphical abnormalities were consistent with active tuberculosis and laboratory confirmation of HIV infection and decision by a clinician to treat with a full course of anti tuberculosis chemotherapy. **Extra-pulmonary tuberculosis** diagnosed if one specimen from an extra-pulmonary site culture-positive for *mycobacterium tuberculosis* or smear-positive for AFB **or** histological or strong clinical evidence consistent with active extra-pulmonary tuberculosis **and** laboratory confirmation of HIV infection **or** strong clinical evidence of HIV infection **and** decision by a clinician to treat with a full course of anti tuberculosis chemotherapy

People living with HIV who were ≥18 years of age and started ART treatment and have follow up at the study sites were included in this study. People lining with HIV who presented with TB before commencing ART, taking TB therapy at the time of HAART initiation, not on ART or discontinued, and severely ill were excluded.

### Sample size determination

The sample size was calculated using Epi Info version 3.5.1 software (Center for Disease Control and Prevention, Atlanta, 2004) using the following parameters: proportion of CD4<50 cells/µl of 31.8% among the controls and 43.9% among cases [32], 5% significance level, power of 80%, a case to control ratio of 1∶2 and by using the two proportion formula. The calculated sample size was 186 for cases and 372 for controls, adding 10% for none response, the resulting minimum sample size was 613 (204 cases and 409 controls). Sample size was calculated for exposure status in different variables of the most significant predictors of TB. First the sample size was calculated for exposure status in different variables; body mass index (BMI<18 kg/m^2^), CD4<50 cell/µl, and low Hgb level. We took the largest sample among these most significant predictors of TB in most literatures that is CD4 cell count less than 50 cell/µl as exposure variable.

### Sampling Technique/Procedure

First, the governmental hospitals and health centers were assessed whether they have adequate cases or not. Two hospitals and thirteen health centers were found to be eligible and included in the study purposely to get adequate number of cases. Identification of cases and controls was done by the principal investigator through the help of the ART and TB registries. All TB–HIV patients on ART who were on anti TB treatment (cases) and fulfilled inclusion criteria were included in the study for their relative small number. Since controls were adequate enough to be sampled, they were selected by simple random sampling method. For those controls that fulfill inclusion criteria, unique identification numbers were given in increasing order by using the registries. Then simple random sampling technique was employed to select samples from each facility. Controls were allocated and selected from each facility based on the number of cases available in each facility with the control to case ratio of 2∶1. I.e. for each case two controls.

### Data Collection and analysis

The data were collected by trained nurses using structured questionnaire, which was translated into Amharic from English, back translated and pre-tested for consistency. The data were collected from two sources: the primary data collected by face to face interview of patients to asses: Socio demographical variables, (age, sex, religion, ethnicity, marital status, employment and educational status), use of substances such as smoking, alcohol and Chat/Khat, medical history like presence of asthma and history of diabetes mellitus, contact history with a TB patient in the family, living conditions (e.g. persons per household (crowding), availability of separate kitchen in the house hold, having latrine in the compound). And to supplement clinical and laboratory information at the ART initiation variables like (CD4 cell count (cells/µL), hemoglobin level mg/dl, WHO clinical stage, functional status, opportunistic infection, chemoprophylaxis) extracted from ART card and log books.

Data were entered and cleaned using Epi-info version 3.5.1 and exported to SPSS software version 16 for analysis. Frequencies and proportions were used to describe the study population in relation to relevant variables. Bivariate analysis was performed to examine the effect of each variable of interest on the risk of TB. Crude odds ratios (COR) and their 95% confidence intervals (CIs) were estimated using binary logistic regression, with TB as an outcome. To identify confounding factors and to measure the independent effects of each exposure variable on occurrence of tuberculosis, a multivariate logistic regression model was used with the variables having a p-value <0.05 in the bivariate analysis. To decide whether the model adequately describes the data, we used the Hosmer-Lemeshow test which indicates a poor fit if the significance value (p) is less than 0.05 and good fit greater than 0.05. Here, in this study the model adequately fits the data since p-value is 0.78.

### Ethical issues

Ethical clearance was obtained from the Institutional Review Board (IRB) of the Addis Ababa University, College of Health science, School of Public Health and from Addis Ababa City Health Bureau Ethics Review committee. Since there were illiterate study participants, the data collectors inform each respondent and confirmed the willingness of the participants by signing on the informed consent sheet. So that consent was obtained from each study participants and confidentiality assured for all the information provided. Moreover, personal identifiers were not included on questionnaire.

## Results

### Socio-demographic, clinical and immunological characteristics of study participants

Of 613 participants selected, 593 study subjects responded (196 (33%) cases and 397(67%) controls) with over all response rates of 96.7% (96.1% for cases and 97.1% for controls).

The mean and inter quartile range (IQR) for the age of cases were 36.7 and 29–42.75 years, respectively. The corresponding values for controls were 35.7 and 30–40 years. More proportions of case and control patients were in the age group of 30–39; 37.2% and 45.6% respectively. High proportions of women were repoted in both groups; 56.6% (111) in cases and 69.3% (275) in controls respectively. More than three fourth of the patients have completed primary school and above; 83.2% in cases and 80.9% in controls. The majority of subjects; 60.2% in cases and 59.9% in controls were single and widowed/divorced.

Majority of cases; 78.4% (152) were in WHO clinical stage III or IV. In contrary, 55.5% (213) control groups were in WHO clinical stage I or II. During ART initiation 85% of cases and 64.9% of controls did not use INH preventive therapy. Of the total 183 patients in cases; three fourth, 73.2% of patients had CD4+ cell count less than 200 cells/µl. But half, 53.6% of patients in controls had CD4+ cell count less than 200 cells/µl. Among cases; 39% of them had Hgb level less than 10 mg/dl. In contrary, 7.2% of controls had Hgb level less than 10 mg/dl **(** **Table 1** **)**.

**Table 1:** Socio-demographic, clinical and immunological characteristics of study participants in Addis Ababa, 2012.

Variables		Cases n(%)	Controls n(%)	Total n (%)
**Sex**	Male	85(43.4)	122(30.7)	207(34.9)
	Female	111(56.6)	275(69.3)	386(65.1)
**Age**	≥40	70(35.)	120(30.2)	149(25.1)
	30–39	73(37.2)	181(45.6)	254(42.8)
	18–29	53(27)	96(24.2)	190(32.0)
**Education**	No education	33 (16.8)	76 (19.1)	109 (18.4)
	Primary	85 (43.4)	159 (40.1)	244 (41.1)
	Secondary	61 (31.1)	122 (30.7)	183 (30.9)
	Tertiary	17 (8.7)	40 (10.1)	57(9.6)
**Marital status**	Married	78(39.8)	159(40.1)	237(40.0)
	Divorced/Widowed	55(28.1)	145(36.5)	200(33.7)
	Single	63(32.1)	93(23.4)	156(26.3)
**WHO Clinical Stage**	Stage III or IV	152(78.4)	171(44.5)	323(55.88)
	Stage I or II	42(21.6)	213(55.5)	255(44.12)
**INH prophylaxis**	Yes	27(15.0)	137(35.1)	164(28.8)
	No	153 (85.0)	253 (64.9)	406 (71.2)
**CTX prophylaxis**	Yes	164 (87.2)	380 (96.2)	544 (93.8)
	No	24(12.8)	15(3.8)	36(6.2)
**Hgb level (mg/dl)**	<10	73(39.0)	28(7.2)	101(17.6)
	10–12.49	54(28.9)	118(30.4)	172 (29.9)
	> = 12.5	60(32.1)	242(62.4)	302(52.5)
**CD4 cell count (cell/µL)**	≤50	26(13.9)	22(5.6)	48(8.3)
	51–200	112(59.9)	187(47.7)	299(51.6)
	201–349	33(17.6)	109(27.8)	142(24.5)
	≥350	16(8.6)	74(18.9)	90(15.5)

WHO = World Health Organization, INH = Isoniazid, CTX = Cotrimoxazole, Hgb = Hemoglobin.

### Clinical presentation of Tuberculosis in HIV positive persons after ART initiation

Half, 50.5% (99) of the TB patients presented with smear negative pulmonary TB followed by extra pulmonary TB, 31.1% (61) and 18.4% (36) patients had smear positive pulmonary TB.

### Bivariate analysis of factors associated with TB

The bivariate analysis showed that higher proportion of male patients (COR = 1.73; 95% CI: 1.23, 2.46) develop TB compared to female patients. The divorced/widowed (COR = 0.560; 95% CI: 0.36, 0.87) patients were less likely to develop TB compared with unmarried (single) individuals. But educational status and occupation were not associated with occurrence of Tuberculosis **(** **Table 2** **)**.

**Table 2:** Socio-demographic determinant factors for occurrence of TB among people living with HIV after ART initiation: comparison of TB cases and controls by bivariate analysis in Binary logistic regression, Addis Ababa, 2012.

Variables		Cases n(%)	Controls n(%)	COR	95% CI	p-value
**Sex**	Male	85(43.4)	122(30.7)	1.73	1.21, 2.46	0.003*
	Female	111(56.6)	275(69.3)	1		
**Age**	≥40	70(35.)	120(30.2)	1.057	0.68, 1.65	0.81
	30–39	73(37.2)	181(45.6)	0.731	0.47, 1.13	0.15
	18–29	53(27)	96(24.2)	1		
**Education**	No education	33 (16.8)	76 (19.1)	1.02	0.508,2.06	0.952
	Primary	85 (43.4)	159 (40.1)	1.26	0.673,2.35	0.472
	Secondary	61 (31.1)	122 (30.7)	1.18	0.617,2.24	0.622
	Tertiary	17 (8.7)	40 (10.1)	1		
**Marital status**	Married	78(39.8)	159(40.1)	0.72	0.48–1.10	0.131
	Divorced/Widowed	55(28.1)	145(36.5)	0.56	0.36, 0.87	0.011*
	Single	63(32.1)	93(23.4)	1		
**Occupation**	Employed	57(29.1)	125(31.5)	0.892	0.61,1.30	0.55
	Unemployed	139(70.9)	272 (68.5)	1		

* **Significant at α = 0.05**.

The cases are more likely to be smoker (COR = 3.34; 95% CI: 2.087, 5.35), alcohol drinker (COR = 2.39; 95% CI: 1.63, 3.52) as well as chat chewer (COR = 2.31; 95% CI: 1.57, 3.40). But Tuberculosis is not associated with diabetes (COR = 1.893; 95% CI: 0.54, 6.62) and history of asthma (COR = 1.3030; 95% CI: 0.59, 2.87). Patients who lived in other place for at least 6 months were about 1.7 times more likely to develop TB after ART initiation (p = 0.017). In addition, controls were more likely to have separate kitchen (p = 0.032) and latrine (p = 0.02). Using gas/kerosene as a source of energy in house hold associated with increased risk of TB (COR = 2.5; 95% CI: 1.74, 3.61). Those who lived in households having a size of 6–10 members were more likely to develop TB compared with the number of persons in the household between 1 and 5 (COR = 1.914; 95% CI: 1.23, 2.99). Similarly, the number of adults in the household between 6 and 10 were 1.89 times more likely to develop TB than adults in the household between 1 and 5 (*P* = 0.043). But, previous family history of TB, history of imprisoned, living in his/her own or family's house and house floor made of cement or mud did not show significant difference between cases and controls **(** **Table 3** **)**.

**Table 3:** Host and environmental determinant factors for occurrence of TB among HIV patients after ART initiation, Addis Ababa, 2012.

Variables		Cases n(%)	Controls n(%)	COR	95% CI	p-value
**Chat chewing**	Yes	70 (35.7)	77 (19.4)	2.31	1.57, 3.40	<0.0001*
	No	126 (64.3)	320 (80.6)	1		
**Smoking**	Yes	49(25.0)	36(9.1)	3.34	2.087,5.35	<0.0001*
	No	147 (75.0)	361(90.9)	1		
**Alcohol drinking**	Yes	71 (36.2)	76 (19.1)	2.39	1.63, 3.52	<0.0001*
	No	125 (63.8)	321 (80.9)	1		
**TB history**	Yes	63 (32.1)	108 (27.2)	1.08	0.74,1.59	0.68
	No	120 (61.2)	223(56.2)	1		
**Asthma**	Yes	11(5.6)	16(4.0)	1.303	0.59, 2.87	0.511
	No	181(92.3)	343(86.4)	1		
**Diabetes mellitus**	Yes	5 (2.6)	5(1.3)	1.893	0.54, 6.62	0.318
	No	187(95.4)	354(89.2)	1		
**Family Hx of TB**	Yes	33(16.8)	75(18.9)	0.847	0.54, 1.33	0.471
	No	159(81.1)	306(77.1)	1		
**Imprisoned**	Yes	15(7.7)	39(9.8)	0.761	0.41, 1.42	0.389
	No	181(92.3)	358 (90.2)	1		
**Have kitchen**	Yes	109(55.6)	257(64.7)	0.682	0.481,0.97	0.032*
	No	87(44.4%)	140(35.3)	1		
**Owen house**	Yes	52 (26.5)	137 (34.5)	0.685	0.47,1.00	0.05*
	No	144(73.5)	260(65.5)	1		
**Latrine**	Yes	160(81.6)	352(88.7)	0.568	0.35, 0.94	0.02*
	No	36 (18.4)	45 (11.3)	1		
**kerosene as source of energy in HH**	Yes	139(70.9)	196(49.4)	2.501	1.74, 3.61	<0.0001*
	No	57(29.1)	201(50.6)	1		
**Number of people living in HH**	>10	3(1.5)	8(2.0)	0.85	0.22, 3.24	0.81
	6–10	44(22.4)	52(13.1)	1.914	1.23, 2.99	0.004*
	1–5	149(76.0)	337(84.9)	1		
**Numbers of room**	1–2	153(78.5)	299(75.3)	1.194	0.79, 1.80	0.397
	> = 3	42(21.5)	98(24.7)	1		

* **significant at α = 0.05**.

**Hx  =  history**.

HH = Houshold.

Other important predictors for the TB occurrence were base line clinical variables. TB patients are more likely to have baseline WHO clinical stage III or IV (COR = 4.51; 95% CI: 3.032, 6.70). Those study subjects with INH prophylaxis (COR = 0.32; 95% CI: 0.21, 0.52) and cotrimoxazole prophylaxis (COR = 0.27; 95% CI: 0.14, 0.53) were less likely to develop TB. Individuals with hemoglobin level <10 mg/dl were more likely to have TB than individuals with hemoglobin level ≥12.5 mg/dl (COR = 10.5; 95% CI: 6.26, 17.68). Patients who were bedridden (COR = 8.87; 95% CI: 4.91, 16.05) and ambulatory (COR = 17.7; 95% CI: 9.98, 31.39) by their functional status were at increased risk of developing TB compared to working status. Similarly, patients whose CD4 cell count ≤50 cell/µL were more likely to develop TB compared to patients who had ≥350 cell/µL cd4 cell count (COR = 5.47; 95% CI: 2.56, 11.97) **(** **Table 4** **)**.

**Table 4:** Clinical and immunological factors for occurrence of TB among HIV patients after ART initiation: bivariate analysis in Binary logistic regression, Addis Ababa, 2012.

Variables		Cases n(%)	Controls n(%)	COR	95% CI	p-value
**WHO clinical stage**	Stage III orIV	152(78.4)	171(44.5)	4.51	3.032, 6.70	<0.0001*
	Stage I or II	42(21.6)	213(55.5)	1		
**INH prophylaxis**	Yes	27(15.0)	137(35.1)	0.33	0.21, 0.52	<0.0001*
	No	153 (85.0)	253 (64.9)	1		
**CTX prophylaxis**	Yes	164 (87.2)	380 (96.2)	0.27	0.14, 0.53	<0.0001*
	No	24(12.8)	15(3.8)	1		
**Functional status**	Bed ridden	39(20.9)	20(5.1)	8.87	4.91, 16.05	<0.0001*
	Ambulatory	70(37.4)	18(4.6)	17.7	9.98, 31.39	<0.0001*
	Working	78(41.7)	355(90.3)	1		
**Opportunistic infection**	Yes	110(59.8)	91(23.6)	4.80	3.29, 7.00	<0.0001*
	No	74(40.2)	294(76.4)	1		
**ART Regimen**	1b	45 (23.0)	35 (8.8)	3.75	1.96, 7.16	<0.0001*
	1c	33(16.8)	115(29.0)	0.84	0.45, 1.54	0.566
	1d	38(19.4)	49(12.3)	2.26	1.197, 4.26	0.012*
	1e	52(26.5)	109(27.5)	1.39	0.78, 2.48	0.246
	1f	5(2.6)	22(5.5)	0.66	0.23, 1.95	0.454
	1a	23 (11.7)	67 (16.9)	1		
**Hgb level (mg/dl)**	<10	73(39.0)	28(7.2)	10.5	6.26, 17.68	<0.0001*
	10–12.49	54(28.9)	118(30.4)	1.85	1.20, 2.83	<0.0001
	> = 12.5	60(32.1)	242(62.4)	1		
**CD4 count (cell/µL)**	≤50	26(13.9)	22(5.6)	5.47	2.56, 11.97	<0.0001*
	51–200	112(59.9)	187(47.7)	2.77	1.54, 4.99	0.001*
	201–349	33(17.6)	109(27.8)	1.400	0.72, 2.73	0.322
	≥350	16(8.6)	74(18.9)	1		

* **significant at α  =  0.05**.

WHO = World Health Organization, INH = Isoniazid, CTX = Cotrimoxazole, ART = Antiretroviral Therapy, Hgb = Hemoglobin, 1a = Stavudine, lamivudine, neverapine→d4t-3TC-NVP, 1b = Stavudine, lamivudine, efavirenz→d4t-3TC-EFV, 1c = Zidovudine, lamivudine, neverapine→AZT-3TC-NVP,1d = Zidovudine, lamivudine, efavirenz→AZT-3TC-EFV, 1e = TDF, lamivudine, efavirenz→TDF-3TC-EFV, 1f = TDF, lamivudine, neverapine→TDF-3TC-NVP.

### Multivariate analysis: Factors independently associated with active TB

To identify independent predictors of developing tuberculosis, a multivariate logistic regression model was fitted with the variables having a p-value <0.05 in the bivariate analysis. So, some variables remained independent predictors for the occurrence of TB after controlling for the other factors. From these factors, being widowed or divorced were at lower risk of TB compared to single individuals (AOR = 0.36; 95% CI: 0.16, 0.82). Patients who had separate kitchen were less likely to have TB (AOR = 0.5; 95% CI: 0.26, 0.96; P<0.038). Presence of INH prophylaxis (AOR = 0.35; 95% CI: 0.125, 0.69; P = 0.005) and cotrimoxazole prophylaxis (AOR = 0.19; 95% CI: 0.06, 0.62) had an independent protective benefit against tuberculosis. Study subjects who were bedridden (AOR = 9.36; 95% CI: 3.39, 25.85) and ambulatory (AOR = 19.4; 95% CI: 7.44, 50.78) by their functional status were more likely to develop TB compared to working status. Study subjects with baseline WHO clinical stage III or IV had higher risk of developing TB (AOR = 3.4; 95% CI: 1.69, 6.87). As well individuals with hemoglobin level <10 mg/dl are more likely to develop TB than individuals with hemoglobin level ≥12.5 mg/dl (AOR = 7.43; 95% CI: 3.04, 18.31). Having opportunistic infection at ART initiation (AOR = 5.22; 95% CI: 2.67, 10.27), the ART regimen initiated at base line and using gas (kerosene) as energy source in the house hold (AOR = 2.67; 95% CI: 1.36, 5.2) were independently associated with increased risk of TB occurrence. But occupational status, smoking, alcohol intake, family history of TB, sex, lived other place, number of people living in the house hold and CD4 cell count lost their statistical significance in the multivariate analysis **(** **Table 5** **)**.

**Table 5:** Factors independently associated with active tuberculosis among HIV infected patients after ART initiation, Addis Ababa, 2012.

Variables		COR(95% CI)	p-value	AOR(95% CI)	p-value
**Kerosene as source of energy in HH**	Yes	2.5(1.7, 3.6)	<0.0001	2.67 (1.36, 5.2)	0.004
	No	1		1	
**Separate kitchen**	Yes	0.68(0.48,0.9)	0.032	0.50 (0.26,0.96)	0.038
	No	1		1	
**WHO clinical stage**	Stage IIIorIV	4.508 (3.03,6.7)	<0.0001	3.40 (1.69, 6.87)	0.001
	Stage IorII	1		1	
**INH preventive therapy**	Yes	0.33(0.21,0.20)	<0.0001	0.35 (0.125,0.69)	0.005
	No	1		1	
**CTX preventive therapy**	Yes	0.27(0.14,0.53)	<0.0001	0.19 (0.06, 0.62)	0.006
	No	1		1	
**Functional status**	Bed ridden	8.88(4.91,16.05)	<0.0001	9.36(3.39, 25.85)	<0.0001
	Ambulatory	17.8 (10,31.4)	<0.0001	19.44(7.44,50.78)	<0.0001
	Working	1		1	
**Opportunistic infection**	Yes	4.80(3.29, 7.0)	<0.0001	5.22 (2.67, 10.27)	<0.0001
	No	1		1	
**Hgb level**	<10	10.52(6.26,17.7)	<0.0001	7.43(3.04, 18.31)	<0.0001
	10–12.49	1.85 (1.2, 2.83)	<0.0001	1.34 (0.65, 2.77)	0.430
	≥12.5	1		1	
**Marital status**	Married	0.72 (0.48,1.1)	0.131	0.99 (0.48, 2.00)	0.985
	Divorced/widowed	0.56(0.36,0.87)	0.011	0.36 (0.16, 0.82)	0.015
	Single	1		1	

## Discussion

This case-control study has identified several determinant factors for the occurrence of TB among HIV infected people enrolled on ART in Addis Ababa. Housing condition, living standard and isoniazid preventive therapy were risk factors for TB in this setting. Patients who have advanced condition (WHO clinical stage III or IV disease, being bedridden and having hemoglobin level less than 10 mg/dl) were also associated with development of new TB infection.

In this study, among determinant factors, marital status was significantly associated with TB. Divorced or widowed Patients were less likely to develop TB compared to unmarried (single), which is consistent with other reports in West Africa and Ethiopia [17], [33]. It might be explained by unmarried (single) persons are younger than married persons and have a different lifestyle, especially males, who often migrate to towns in search of a job where they live alone or with friends.

Similarly, low level of education was not associated with TB. But it is not consistent with the study from south west Ethiopia [16]. This could be due to the high prevalence of literates in our study population as it was from the capital of the country.

Smoking was identified as risk factor for the development of TB in clinic-based case-control study in West Africa [17]. But, in a case control study in Gambia, smoking was not associated with active TB [18]. Similarly, in this study smoking was not associated with TB occurrence in multivariate analysis. This could be due to the low prevalence of smoking in our study population. There could also be a social desirability bias whereby smokers denied their smoking status.

In a case control study from West Africa, history of asthma became protective against TB [17]. But, in this study history of asthma was not associated with TB. This is consistent with the case control study in Gambia [18].

Studies demonstrated that contact with TB patients in the family associated with increased occurrence of TB [16]–[18]. But in this study family history of TB showed some degree of association with TB in bivariate analysis, but it did not have an independent effect on the occurrence TB in multivariate analysis when adjusted for other variables. This could be due to that the influence of TB history in the family as a risk factor for TB would differs by setting and background of HIV burden.

Other independent predictors of tuberculosis were WHO stage III or IV; patients with WHO stage III or IV have higher risk of developing TB than those with WHO stage I or II. It is consistent with other studies done in South Africa and South West Ethiopia [16], [23]. This suggests that who had WHO stage III or IV might be immune-compromised and predisposed to TB.

TB patients were 8.87 times more likely to be bedridden at the initiation of ART than working patients. This is consistent with the retrospective cohort study in Ethiopia [33]. High degree of suspicion while administering ART towards patients with this condition should be instituted.

Besides, availability of separate kitchen in the household associated with decreased risk of TB development which is consistent with a study from south west Ethiopia [16]. It might be explained by increased indoor air pollution if there is no separate cooking kitchen in the house. Likewise using gas (kerosene) as energy source in the household associated with TB as it was commonly used cooking fuels in urban areas.

Studies have shown that risk of TB was associated with the number of people living together in the household (over Crowding) [18], [19], [21]. But, this study did not find the association between TB and number of people in the household. This might be related to high proportions of unmarried persons in the study population resulted in low number of family size in the house hold. The other reason may be due to TB development is a reactivation of an infection acquired years ago due to HIV infection, with no relation to current living and crowding conditions.

In addition, patients having a hemoglobin level of ≤10 mg/dl have 2.4 times higher risk of developing TB than those patients having hemoglobin level ≥12.5 mg/dl, similar to other study findings in south west Ethiopia [16]. This shows that patients having higher hemoglobin level were less likely to develop TB than those with low hemoglobin level. TB and hemoglobin level might be indirectly associated with advanced stage of HIV disease. When HIV positive patients have chronic disease and high viral load, it resulted in immune-suppression and suppression of red blood production in bone marrow. This is also consistent with the previous findings that predict the occurrence of TB which implied that advance disease condition in HIV patients may predict occurrence of Tuberculosis after ART initiation.

Different studies have shown that isoniazed (INH) preventive therapy reduces the risk of TB infection in people living with HIV [24]–[27]. Similarly, in this study, patients who were on INH preventive therapy were at the lower risk of developing TB. The initiation of cotrimoxazole preventive therapy has also been independent predictor. TB/HIV collaborative actions should give priority high level of coverage to the implementation of these interventions as they have proven effectiveness in improving patients' conditions.

A Study from West Africa showed that ownership of the house by the TB patient's family associated with lower risk for TB [17]. But, this study didn't show statistical difference between those who had house and those who hadn't. This inconsistency might be due to source population difference, as the source population of this study was from the capital of the country.

This study has the following limitations: Case control study design could not set up temporal relationships and can only show associations; it could not proof causations. Recall bias might have also affected the accuracy of information related to substance use such as cigarette smoking and alcohol consumption. Lastly, challenges to diagnosis of tuberculosis in HIV patients might result in low sensitivity and specificity of available diagnostic approaches.

## Conclusion

Having poor clinical and biochemical status were found to be predicators of occurrence of Tuberculosis. All people living with HIV/AIDS should be screened for TB. But, in the presence of the risk factors mentioned in this paper, intensified screening is highly recommended during follow up of treatment. In addition, increasing coverage of INH and cotrimoxazole preventive therapy is necessary to reduce the overall risk of TB among HIV patients who started ART. Household condition related to kerosene use in household was also associated with outcome of interest.
